# Comorbid Conditions in Patients With Metastatic Colorectal Cancer

**DOI:** 10.4021/wjon370e

**Published:** 2011-10-28

**Authors:** Alex Z Fu, Zhongyun Zhao, Sue Gao, Beth Barber, Gordon G Liu

**Affiliations:** a Department of Quantitative Health Sciences, Cleveland Clinic, Cleveland, Ohio, USA; b Amgen Inc, One Amgen Center Drive, Thousand Oaks, CA 91320-1799, USA; c Guanghua School of Management, Peking University, Beijing, China

**Keywords:** Cardiovascular disease, Comorbidities, Metastatic colorectal cancer, Patient management

## Abstract

**Background:**

Patients with metastatic colorectal cancer (mCRC) often have other medical conditions that may impact treatment decisions, prognoses and quality of care. We aimed to assess co-existing medical conditions in the mCRC patient population. This retrospective cohort study used linked medical and pharmacy claims data from two US-based Medstat MarketScan claims databases and identified patients with newly diagnosed mCRC between January 2005 and June 2008.

**Methods:**

Patient data were analyzed for comorbid conditions and medication use in the year prior to diagnosis of mCRC. Univariate analyses were conducted to compare the comorbid conditions between patients aged ≥ 65 and < 65 years old. In total, 12 648 patients aged ≥ 18 years were identified. The study was evenly populated by gender and age above and below 65, and most patients had a primary diagnosis of colon cancer (70.1%).

**Results:**

The most prevalent comorbidity was cardiovascular disease (CVD) (55.7% of patients) including hypertension (40.8%), cardiac dysrhythmia (14.2%), coronary artery disease (13.5%), congestive heart failure (7.2%) and arterial and venous thromboembolism (6.2% and 4.6%, respectively). Most comorbidities were significantly more prevalent in patients ≥ 65 years of age, particularly with respect to CVD (67.9% versus 42.5%, respectively; P < 0.0001). Additionally, nearly half (49.7%) of the patients received antihypertensive agents and many patients were prescribed more than one class of medications prior to mCRC diagnosis.

**Conclusions:**

Comorbid medical conditions, particularly CVDs, are common in patients with mCRC, which could increase the complexity of patient management. This should be a consideration integral to the selection of the most appropriate treatment for individual patients.

## Introduction

Colorectal cancer (CRC) is the second leading cause of death from cancer in the USA, with 146 970 new cases and 49 920 deaths estimated in 2008 [1]. Of newly diagnosed CRC patients, 15% to 25% have metastatic disease at diagnosis, while disease recurrence and the development of distant metastases occur in up to 50% of all patients initially diagnosed at earlier disease stages [2]. Similarly in Europe, CRC is also the second most common form of cancer and the second most common cause of death from cancer [3].

Overall survival for patients with metastatic CRC (mCRC) has increased dramatically in the last 20 years largely due to advances in systemic therapy (newer chemotherapies and the introduction of biologic agents) [4]. Additionally, the treatment has become more personalized for patients with mCRC. For example, the benefits of the epidermal growth factor receptor (EGFR) antagonists cetuximab and panitumumab are limited to patients with wild-type KRAS (the proto-oncogene Kirsten ras sarcoma virus) mCRC. Although there are significant gains in clinical benefit, biologics are associated with recognized adverse events (AEs) that may limit their beneficial effects in some patients [5-7]. Gastrointestinal perforations, fistulae, haemorrhage, hypertension and arterial thromboembolism are some of the serious AEs associated with bevacizumab [8, 9], while the EGFR antagonist class is associated with hypomagnesaemia, infusion reactions and skin toxicities [7, 10-14].

The incorporation of the three different biologic treatments into the mCRC armamentarium offers a degree of flexibility regarding the most appropriate choice of biologics. Consideration of AE risk plays a role in treatment selection to ensure an acceptable risk–benefit profile. Pre-existing comorbidities in patients with mCRC may also play a role in order to avoid toxicity issues [15]. Taking age and comorbidities into consideration as part of treatment selection is, therefore, not uncommon in the management of cancer. For example, in advanced lung cancer, both age and comorbidity play an important role in treatment decisions [16]. There are, however, limited existing data regarding the extent of comorbid conditions in patients with mCRC.

The aim of this study was to comprehensively assess co-existing medical conditions in the mCRC patient population in clinical practice.

## Methods

### Source data

This was a retrospective cohort study using longitudinal, integrated medical and pharmacy claims data from two Medstat MarketScan claims databases: the Commercial Claims and Encounters database and the Medicare Supplemental and Coordination of Benefits database. These databases include fully de-identified patient-level, paid and adjudicated medical and pharmacy claims histories of 30 million covered lives belonging to 12 national and regional health plans in the USA. The databases are representative of the US national commercially-insured population and those who have both Medicare coverage and supplemental employer-sponsored coverage. They capture the full continuum of care in all settings including physician office visits, hospital stays and outpatient pharmacy claims.

### Sample selection and data extraction

Data on patients with newly diagnosed mCRC between January 2005 and June 2008 were extracted from the databases using the International Classification of Disease 9th Revision, Clinical Modification (ICD-9-CM) diagnosis codes for CRC (153.x [excluding 153.5], 154.0, 154.1, 154.8) and distant metastasis (196.0, 196.1, 196.3, 196.5, 197.x [excluding 197.5], 198, 199.0). The index date was defined as the date of the initial mCRC diagnosis. Only patients aged ≥18 years at the index date and with at least 1-year continuous medical and drug benefit coverage prior to the index date, and with a first identified distant metastasis diagnosis date no more than 30 days after the first identified CRC diagnosis date were included in the data set. Demographic and clinical characteristics reported included age, gender, geographical location, type of insurance plan, cancer type (colon versus rectal) and location of metastases.

Comprehensive comorbidities were examined in this study including cardiovascular disease (CVD); existing wounds (bone fractures, wound-healing complications, open wounds); history of bleeding/haemorrhage; digestive system disorders; diabetes mellitus; diseases of the blood; diseases of the skin; respiratory system disorders; smoking history; renal failure; and obesity. Using ICD-9 diagnosis codes, comorbid medical conditions were examined during the 1-year prior to the index date, and in the case of traumatic conditions (e.g., bone fracture and open wound) data during 30 days prior to the index date were assessed. Additionally, data on medications taken during the 1 year prior to the index date were extracted using prescription information.

### Statistical analysis

Patient demographics and comorbid conditions defined by ICD-9 codes prior to mCRC diagnosis were summarized descriptively for the overall patient population using the 1-year pre-index period data. Similarly, medications received prior to mCRC diagnosis were summarized descriptively. Univariate analyses were conducted to compare the comorbid conditions between patients aged ≥ 65 and < 65 years old. Chi-square tests were used to compare proportions of comorbidities between patients aged ≥ 65 and < 65 years old.

## Results

Based on the selection criteria, 12 648 patients were eligible for inclusion in the analysis of comorbid conditions and medication use in the year prior to diagnosis of mCRC. The study population had 54% of men, and just over half were aged > 65 years (52%). The majority of the study population had a primary diagnosis of colon cancer (70%) as opposed to rectal cancer, and the mean age of the study population (standard deviation) was 66.3 (13.1) years (Table 1).

**Table 1:** Characteristics of Study Population

Variable	n	Mean or percentage
Age, mean ± SD, year	12 648	66.3 ± 13.1
18 ≤ Age < 40	240	1.9%
40 ≤ Age < 50	1012	8.0%
50 ≤ Age < 65	4819	38.1%
Age ≥ 65	6577	52.0%
Gender: male (versus female)	6855	54.2%
Geographic region		
Northeast	1189	9.4%
North Central	4364	34.5%
South	4490	35.5%
West	2568	20.3%
Unknown	38	0.3%
Urban versus rural residence		
Urban	10 245	81.0%
Rural	2353	18.6%
Missing	51	0.4%
Insurance plan type		
Comprehensive	4174	33.0%
HMO	2125	16.8%
PPO	5059	40.0%
POS-non-capitated	734	5.8%
Other/unknown	557	4.4%
Cancer type: colon (versus rectum)	8866	70.1%
Location of metastasis		
Liver	5084	40.2%
Lung	1796	14.2%
Bone	696	5.5%
Brain	379	3.0%
Others	4693	37.1%

SD: standard deviation; HMO: Health Maintenance Organization; PPO: Preferred Provider Organization; POS: Point of Service plan.

The most frequent comorbidities in the overall population are shown in Table 2, which indicates that CVDs were most commonly reported (55.7%), followed by digestive system disorders (29%), a history of bleeding (28.3%) and diabetes mellitus (19.1%). Among the patients with comorbid CVD, hypertension was the most common condition (40.8%) followed by heart disease (28%), including cardiac dysrhythmia (14.2%), coronary artery disease (13.5%), congestive heart failure (7.2%), ischemic heart disease (6.2%), arterial thromboembolism (ATE) (6.2%), and venous thromboembolism (VTE) (4.6%).

**Table 2:** Comorbid conditions: numbers and percentages (N = 12 648)

Comorbidities*	Total population (n = 12 648)		Age < 65 (n = 6070)		Age ≥ 65 (n = 6578)		p-value (between < 65 and ≥ 65)
	n	%	n	%	n	%	
Cardiovascular disease	7047	55.7	2581	42.5	4466	67.9	< 0.0001
Hypertension	5166	40.8	1990	32.8	3176	48.3	< 0.0001
Heart disease	3544	28.0	945	15.6	2599	39.5	< 0.0001
Coronary artery disease	1711	13.5	404	6.7	1307	19.9	< 0.0001
Acute myocarditis	1	0.0	0	0.0	1	0.0	
Cardiomyopathy	234	1.9	69	1.1	165	2.5	< 0.0001
Cardiac dysrhythmia	1792	14.2	426	7.0	1366	20.8	< 0.0001
Congestive heart failure	905	7.2	174	2.9	731	11.1	< 0.0001
Acute myocardial infarction	215	1.7	58	1.0	157	2.4	< 0.0001
Other ischemic heart disease	785	6.2	232	3.8	553	8.4	< 0.0001
Stroke	497	3.9	108	1.8	389	5.9	< 0.0001
VTE	576	4.6	257	4.2	319	4.8	0.10
ATE	784	6.2	205	3.4	579	8.8	< 0.0001
Diabetes mellitus	2418	19.1	999	16.5	1419	21.6	< 0.0001
Existing wound	2006	15.9	962	15.8	1044	15.9	0.97
Digestive system	3668	29.0	1692	27.9	1976	30.0	0.007
Diseases of blood and blood forming organs	340	2.7	175	2.9	165	2.5	0.19
Diseases of skin and subcutaneous tissue	592	4.7	204	3.4	388	5.9	< 0.0001
Renal failure and insufficiency	989	7.8	348	5.7	641	9.7	< 0.0001
History of bleeding	3574	28.3	1825	30.1	1749	26.6	< 0.0001
Diseases of respiratory system	1974	15.6	629	10.4	1345	20.4	< 0.0001
Smoking history	49	0.4	28	0.5	21	0.3	0.20
Obesity	161	1.3	129	2.1	32	0.5	< 0.0001

*Some patients had more than one type of comorbidity and are tabulated under each condition; VTE: venous thromboembolism; ATE: arterial thromboembolism.

Elderly patients ≥ 65 years of age had a significantly higher prevalence of CVDs compared with younger patients (67.9% versus 42.5% respectively; P < 0.0001; Table 2). Similarly, individual heart-related comorbidities were significantly more prevalent in those aged ≥ 65 years (39.5% versus 15.6%; P < 0.0001) with the exception of acute myocarditis (Table 2). The prevalence of diabetes mellitus was also significantly higher among the older age group (21.6% versus 16.5%; P < 0.0001), as were comorbidities relating to diseases of the skin and subcutaneous tissue, renal failure and insufficiency, and respiratory diseases (P < 0.0001). Obesity and history of bleeding were the only two comorbidities with a significantly higher prevalence in those aged < 65 years of age (P < 0.0001).

Medications received prior to diagnosis are shown in Table 3. The most common medications were antibiotics (61.7%), and antihypertensive agents (49.7%). The percentages in Table 3 cumulatively suggest that many patients were prescribed more than one class of medications prior to mCRC diagnosis.

**Table 3:** Medications Received Prior to Diagnosis of mCRC (N = 12 648)

Variable	Number	Percentage (%)
Cardiac drugs		
Antihypertensives	6286	49.7
Other cardiac drugs	4123	32.6
Anticoagulant	1492	11.8
Antiplatelet drugs	734	5.8
Vasodilating drugs	734	5.8
Antibiotics	7804	61.7
Antihyperlipidemics	3719	29.4
Diuretics	3036	24.0
NSAIDs	2188	17.3
Antiallergic drugs	1986	15.7
Antidiabetic drugs	1935	15.3
Antidiarrhoea drugs	1075	8.5
Anticonvulsants	974	7.7
H2 antagonists	696	5.5
Antiulcer	696	5.5

NSAIDs: non-steroidal anti-inflammatory drugs.

## Discussion

The findings of this retrospective cohort study indicate that comorbid medical conditions are common in patients with mCRC. CVDs are the most prevalent comorbidities, and are significantly more prevalent in patients over 65 years old, affecting more than two-thirds of this group. As might be anticipated based on the comorbidities identified, this study also showed that patients with mCRC are frequently treated with non-CRC-related medications, mostly for CVDs and gastrointestinal diseases. The high frequency of CVD as a comorbidity in this study population might be anticipated given that more than half of patients were > 65 years of age. In addition to hypertension, the most frequently reported CVDs were heart diseases (including cardiac dysrhythmia and congestive heart failure), stroke, ATE and VTE.

Although data on pre-existing comorbidities in mCRC are limited, some information is available on patients with CRC of any stage in whom the most common comorbidities are reported to be similar to those presented here. Yancik et al [17] reported hypertension, serious heart conditions, gastrointestinal problems, arthritis and chronic obstructive pulmonary disease as the most prominent comorbid conditions in patients with colon cancer of any stage and these findings have been corroborated by later studies [18-20]. Our finding showed that many co-existing conditions, including the majority of CVD comorbidities, diabetes mellitus, diseases of the skin and subcutaneous tissue, renal failure and insufficiency, and respiratory diseases, occurred more frequently in patients aged > 65 years is also supported by data from the colon cancer setting [17].

Some of the comorbidities identified in our study are likely to be of particular importance to consider. For example, comorbid conditions relating to a history of bleeding and/or existing wounds were identified in 28.3% and 15.9% of patients in this cohort study, respectively. Wound-healing complications have also been linked with treatment regimens that include bevacizumab [8, 21]. This may be particularly important in patients who are eligible for metastasectomy. In consideration of these factors, a delay in elective surgery of up to 8 weeks after completion of bevacizumab, and a similar delay in restarting after liver surgery, is recommended [22].

Our findings also show that VTE and ATE occurred in 4.6% and 6.2% of patients with mCRC, respectively. A recent meta-analysis evaluating the use of bevacizumab in nearly 8000 patients with a variety of advanced solid tumours from 15 randomized controlled trials (RCTs) concluded that the drug was significantly associated with an increased risk of developing VTE (P < 0.001) compared with controls, an effect independent of dose [23]. An earlier meta-analysis found an association between bevacizumab and ATE (P = 0.031) using data from five RCTs [24].

Comorbid diseases of the skin and subcutaneous tissue together were identified in 4.7% of all patients, increasing in frequency in the elderly sub-population. This is of relevance to the management of mCRC as skin toxicities are frequently reported in clinical trials of cetuximab and panitumumab [7, 11, 14, 25].

Our study is associated with a number of limitations. The comorbidities identified in this study were based on healthcare service use data, thus comorbid conditions that did not trigger healthcare service use in the year prior to mCRC diagnosis were not captured. Also, the prevalence rates for conditions of smoking history and obesity identified in this study were very low. This is likely due to under-reporting of these two conditions in claims databases. Finally, our results are reflective of the population studied and may not be extrapolated to the mCRC population in general.

In conclusion, comorbid conditions were frequently observed in patients prior to a diagnosis of mCRC. The presence of these comorbidities increases the complexity of managing the patient’s condition. In current treatment guidelines, the presence of comorbidities is included as a factor that should be considered in the choice of chemotherapy for mCRC [15]. Given that, in addition to chemotherapy, there is now a choice of biologic agents for mCRC, each with a well-defined and distinct AE profile, consideration of comorbid conditions should now be integral to the selection of all components of the treatment regimen for individual patients with mCRC.
